# Structural and Ultrastructural Characteristics of Bone-Tendon Junction of the Calcaneal Tendon of Adult and Elderly Wistar Rats

**DOI:** 10.1371/journal.pone.0153568

**Published:** 2016-04-14

**Authors:** Diego Pulzatto Cury, Fernando José Dias, Maria Angélica Miglino, Ii-sei Watanabe

**Affiliations:** 1 Department of Anatomy, Institute of Biomedical Sciences, University of São Paulo, São Paulo, Brazil; 2 Department of Surgery, School of Veterinary Medicine and Animal Science, University of São Paulo, São Paulo, Brazil; 3 Department of Surgery and Anatomy, School of Medicine of Ribeirão Preto, University of São Paulo, Ribeirão Preto, Brazil; 4 CICO Research Centre, Dental School, Universidad de La Frontera, Temuco, Chile; Université de Lyon—Université Jean Monnet, FRANCE

## Abstract

Tendons are transition tissues that transfer the contractile forces generated by the muscles to the bones, allowing movement. The region where the tendon attaches to the bone is called bone-tendon junction or enthesis and may be classified as fibrous or fibrocartilaginous. This study aims to analyze the collagen fibers and the cells present in the bone-tendon junction using light microscopy and ultrastructural techniques as scanning electron microscopy and transmission electron microscopy. Forty male Wistar rats were used in the experiment, being 20 adult rats at 4 months-old and 20 elderly rats at 20 months-old. The hind limbs of the rats were removed, dissected and prepared to light microscopy, transmission electron microscopy and scanning electron microscopy. The aging process showed changes in the collagen fibrils, with a predominance of type III fibers in the elderly group, in addition to a decrease in the amount of the fibrocartilage cells, fewer and shorter cytoplasmic processes and a decreased synthetic capacity due to degradation of the organelles involved in synthesis.

## Introduction

The calcaneal tendon, also known as Achilles tendon, is the thickest and strongest tendon in the human body [1]. Tendons are typically described as a dense fibrous connective tissue that attaches the muscles to the bones. It is constituted by a large density of collagen fibers and a small number of cells, what produces extremely high tensile forces [2].

The collagen fibers are wrapped by a layer of connective tissue known as endotenon that contains blood vessels, lymphatics, and nerves, to form higher structural units called fascicles, which are surrounded by another connective tissue layer, epitenon, to form the tendon [3].

The main function of the tendons is to transfer the contractile forces generated by the muscles to the bones, generating movement. The region where the tendons attach to the bones is called bone-tendon junction or enthesis [4,5].

The entheses may be classified as fibrous or fibrocartilaginous [6]. In the fibrous entheses, tendons and/or ligaments attach to the shaft (diaphysis) of the long bones and in the fibrocartilaginous, the attachment occurs at the epiphyses of the long or short bones [2].

The attachment of the tendon to the bone constitutes a complex transition area of about 1mm [7]. This whole area is composed of 4 zones that are better identified by light microscopy with picrosirius red staining under polarized light. These areas are: 1) tendon, 2) uncalcified fibrocartilage, 3) calcified fibrocartilage, 4) bone.

The first zone is the terminal part of the tendon. Its lamellar tissue is composed of collagen bundles longitudinally aligned, separated by loose connective tissue that merges into the peritenon and contains a variable number of elastic fibers. The tendon changes gradually over a distance of a few microns into the second zone that is the uncalcified fibrocartilage. The cells take on the chondrocyte phenotype, becoming rounded and arranging themselves in pairs or rows within the lacuna. The third zone is composed of calcified fibrocartilage, the passage from the second to the third zone occurs abruptly at a mineralization front seen as a basophilic line (tidemark). Finally, the fourth zone is composed of trabecular bone [8–10].

The elderly population is growing in number worldwide remaining more physically active and increasingly susceptible to injury. It is estimated that by 2030, 70 million people in the United States will be over the age of 65 [11] and by 2020 in Brazil about 26.3 million people, representing 12.9% of the total population [12].

Aging is the biggest risk factor for tendon disorders. Age-related changes affecting structural and mechanical levels may predispose tendons to injury [13].

The tendon is subjected to early degenerative changes, since both the collagen and noncollagenous matrix components of tendons show qualitative and quantitative changes and these process may be detected as early as the third decade [14,15].

The calcaneal tendon was chosen for this study because its ruptures rates are one of the most common tendon injuries in the adult population as reported by [16,17], as well as the incidence of acute calcaneal tendon rupture has increased from 1994 to 2013 as a result of increasing incidence in the older population [18].

In terms of bone, aging itself is an effective predictor of osteoarthritis, bone loss, development of osteoporosis and fracture [19].

The aim of this study is to contribute to the knowledge of the changes in the bone-tendon junction of the calcaneal tendon that result from the aging process. Thus, this study compared the structural and ultrastructural aspects of the bone-tendon junction of calcaneal tendon of adults and elderly Wistar rats.

## Materials and Methods

Forty male Wistar rats *(Rattus norvegicus)* were obtained from the animal house of the Institute of Biomedical Sciences of the University of São Paulo. Rats were organized organized in two groups, 20 adults rats (4 months-old) and 20 elderly rats (20 months-old). Each group was further divided in subgroups of five rats that were prepared to light microscopy [five to hematoxylin-eosin (HE) and five to picrosirius red], scanning electron microscopy and transmission electron microscopy, we used both ankles in all techniques. All rats were maintained in plastic standard cages (5 rats in each cage) at the animal house of the Institute of Biomedical Sciences of the University of São Paulo. The rats had free access to food and water and were raised under a controlled room temperature (23 ± 1°C) and a light–dark cycle of 12 h. The rats were euthanized with an overdose of Ketamine (150 mg/kg) and Xylazine (10 mg/kg) [20].

The experiment was conducted in accordance to the Ethical Principles of Animal Experimentation adopted by the Brazilian School of Animal Experimentation (COBEA) and was approved by the Animal Experimentation Ethics Committee (EAEC) of the School of Veterinary Medicine and Animal Science of the University of São Paulo (process no. 2536/2012) and the Animal Experimentation Ethics Committee of the Institute of Biomedical Science of the University of São Paulo (process no. 120/12).

The experiments were conducted at the Anatomy Department of the Institute of Biomedical Sciences of the University of São Paulo.

### Histological Procedures

The ankles of rats were removed and immersed in 4% formaldehyde fixative solution (Synth #01P1005.01.AF, Diadema-SP, Brazil) for 48h at room temperature. After fixation of the tissues, the samples were rinsed with distilled water and dissected. The samples were subsequently immersed in 7% EDTA Disodium Salt (Synth #E2005, Diadema-SP, Brazil) demineralizing solution until complete demineralization with replacement of the EDTA solution 3 times a week.

After the samples were dehydrated in a graded ethanol series (70–100°, 30 min for each grade) the samples were diaphanized immersing the sample in xylene (Synth #00X1001.14.BJ, Diadema-SP, Brazil) for 30 min in three repetitions. Thus, the samples were embedded in paraffin for microtome sectioning.

Sections of 6 μm thickness were mounted in glass slides and stained with HE protocol: The slides were immersed in hematoxilin solution (Sigma-Aldrich #HHS16, São Paulo-SP, Brazil) for 3 min, washed in tap water for 5 min, transferred to eosin (Sigma-Aldrich #E4009, São Paulo-SP, Brazil) for 1 min and rinsed in tap water. We dehydrated the samples in graded ethanol series (95–100°, 2 min each), xylene 3 times 2 min each and coverslipped with Entellan^®^ (Merck #107961, Darmstadt-HE, Germany).

The picrosirius red protocol, briefly consisted in immersion of slides in picrosirius red solution (EasyPath # EP-11-20011, São Paulo-SP, Brazil) for 60 min, rinsed in tap water, dehydrated and coverslipped as described above. Conventional and polarized histological images were analyzed with a light microscope Nikon Eclipse E600 equipped with a Nikon Digital Sight DS-Ri1 camera.

To polarized images microscope was configured and all images were then obtained only once to avoid configuration interactions, because microscope stage rotation may change the color of the collagen fibers [21].

Macroscopic view from region analyzed is in the S1 Fig.

### Scanning Electron Microscopy (SEM) Procedures

The samples were removed after perfusion with a modified Karnovsky solution containing 2.5% glutaraldehyde (Sigma-Aldrich #V000383, São Paulo-SP, Brazil) and 2% formaldehyde (Synth #01P1005.01.AF, Diadema-SP, Brazil) in 0.1 M sodium phosphate buffer at pH 7.4 (Synth #F1034.01.AH plus #F1033.01.AF, Diadema-SP, Brazil) [22]. The tissues were then immersed in the same solution for 24h at 4°C, rinsed in 0.1 M phosphate buffer solution at pH 7.4. The samples were cryofractured [23], rinsed again in sodium phosphate buffer solution, postfixation in 1% osmium tetroxide (EMS #19100, Hatfield-PA, USA) for 2h at 4°C, rinsed with distilled water and dehydrated in a graded series of alcohol (70 to 100%, 15 min each grade). The samples were dried in Balzers CPD-030 critical point apparatus using liquid CO_2_, and mounted in metal stubs and coated with gold ions in Balzers Union SCD-040 apparatus.

The samples were analyzed in the scanning electron microscope Jeol JSM-7401F of the Analytical Center of the Institute of Chemistry of the University of São Paulo.

Macroscopic view from region analyzed is in the S1 Fig.

### Transmission Electron Microscopy (TEM) Procedures

The samples were dissected after perfusion and immersed in 2.5% glutaraldehyde (Sigma-Aldrich #V000383, São Paulo-SP, Brazil) for 4h at room temperature. Demineralized with 1.9% glutaraldehyde, 0.06 M sodium cacodylate buffer (EMS, #12310, Hatfield-PA, USA) and 0.15 M EDTA at pH 7.4 (Synth #E2005, Diadema-SP, Brazil) [24]. They were then rinsed in phosphate buffer solution, postfixation with 1% osmium tetroxide at 4°C for 2h. The samples were dehydrated in a graded series of alcohol (30–100%, 30 min each grade) followed by 2 stages of dehydration in propylene oxide (EMS, # 20401, Hatfield-PA, USA) for the same time interval and embedded in Spurr’s resin^®^ (EMS, #14300, Hatfield-PA, USA). Ultrathin sections (thickness 65 nm) were obtained using diamond knives in a Reichert Ultra Cut microtome, collected on copper grids (200-mesh) (EMS, #G200-Cu, Hatfield-PA, USA) [22]. The grids were counterstained with 4% uranyl acetate (EMS, #22400, Hatfield-PA, USA) [25] and 0.4% lead citrate solutions (Sodium Citrate Synth #01C1033.01.AF, Diadema-SP, Brazil plus Lead Nitrate Sigma-Aldrich #V000361, São Paulo-SP, Brazil) [26] and examined in a Jeol JEM 1010 at 80 kV at the Microscopy Center of the Department of Development and Cell Biology of the Institute of Biomedical Sciences of the University of São Paulo.

Macroscopic view from region analyzed is in the S1 Fig.

### Statistical Analyses

The statistical significance of the results were assessed with unpaired Student's T-Test with Welch’s correction for two independent groups using the software GraphPad Prism 5 for *p* < 0.05. The normality of the data was assessed using the Shapiro-Wilk test, thus permitting the use of parametric tests. Data are presented as mean ± standard deviation [27]. To this technique were used both hind limbs of each rats.

#### Collagen Quantification

To quantify collagen types I and III in the area of bone-tendon junction of the calcaneal tendon we used histological images stained by the method of picrosirius red and analyzed under a polarized light microscope. The utilization of this method was certified by Dapson and colleagues (2011) [28].

Each photomicrograph is composed by three monochromatic layers—yellow, red and green, collagen type I is stained in yellow and red and type III in green [29,30]. The specificity and applicability of this method was explained since the 70’s [31]. The images were processed with Photoshop CS6, after the decomposition of the photomicrographs into the three layers, they are converted into binary images and the collagen is quantified. This step was conducted with ImageJ 1.47v [32]. The region used to quantify collagen in both groups may be seen in S2 Fig.

#### Fibrocartilage Cells and Insertion Thickness

To evaluate the amount of fibrocartilage cells present in uncalcified and calcified fibrocartilage regions we used histological images stained with hematoxylin-eosin. A 0.15 mm^2^ rectangle was superimposed on the entire enthesis region. The rectangle was divided into two equal parts and the dividing line was placed over the tidemark, thus creating two regions of fibrocartilage with equal area. Rats from adult group presenting double tidemark the dividing line was placed between each tidemark. Only the cells contained in the rectangle were counted (S3 Fig).

Insertion thickness of the tendon was measured near the end of the uncalcified fibrocartilage (S4 Fig).

## Results

### Light Microscopy

**Fig 1:**
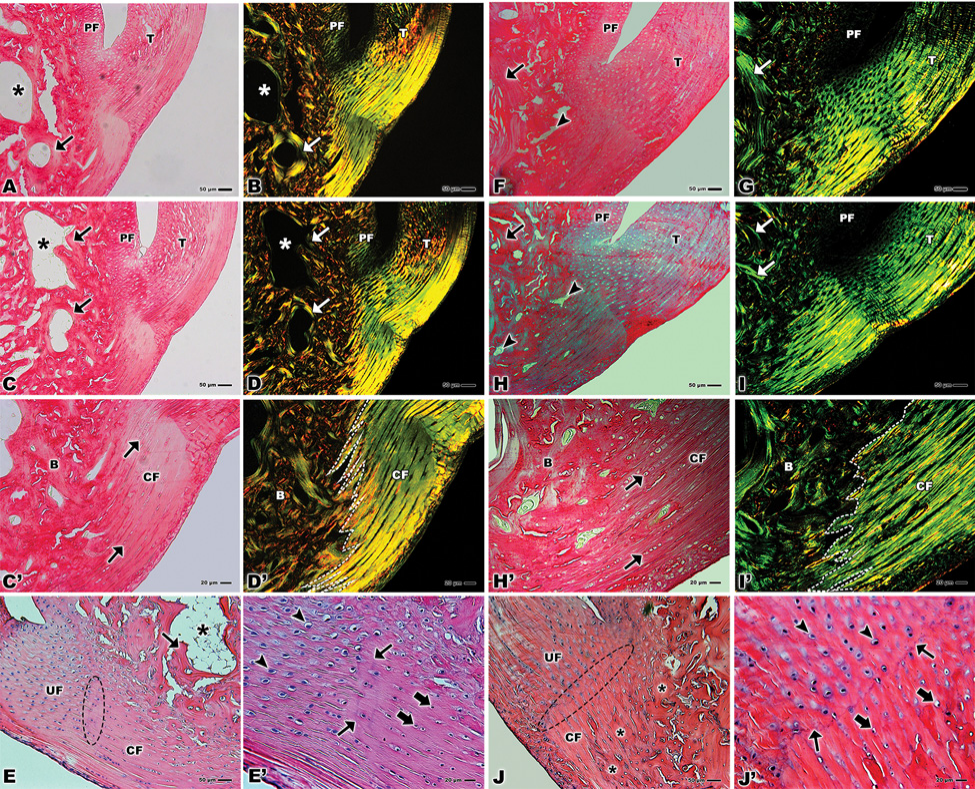
Light microscopy of bone-tendon junction of the calcaneal tendon of adults Wistar rats (A, B, C, D, E, C’, D’, and E’) and elderly Wistar rats (F, G, H, I, J, H’, I’, and J’). **(A, B, C, D)** Calcaneal tendon (T), periosteal fibrocartilage (PF), trabecular space (*), trabecular bone (arrows). Stain: Fig A: Picro-Sirius. Fig C: Picro-Sirius under polarized light. Bars: 50 μm, x100. **(C’)** Calcified fibrocartilage (CF), rows of cells (arrows), bone (B). Stain: Picro-Sirius. Bar: 20 μm, x200. **(D’)** Calcified fibrocartilage (CF), rows of cells (arrows), bone (B), division between the collagen fibers of the tendon and bone (dashed line). Stain: Picro-Sirius under polarized light. Bar: 20 μm, x200. **(E)** Uncalcified fibrocartilage (UF), calcified fibrocartilage (CF), tidemark (dashed ellipse), trabecular space (*), trabecular bone (arrow). Stain: Hematoxylin–eosin. Bar: 50 μm, x100. **(E’)** Fibrocartilage cells in the uncalcified fibrocartilage (arrowhead), fibrocartilage cells in the calcified fibrocartilage (larger arrows), tidemark (small arrows). Stain: Hematoxylin–eosin. Bar: 20 μm, x200. **(F, H)** Calcaneal tendon (T), trabecular space (arrowhead), trabecular bone (arrow), periosteal fibrocartilage (PF). Stain: Picro-Sirius. Bar: 50 μm, x100. **(G, I)** Calcaneal tendon (T), trabecular bone (arrows), periosteal fibrocartilage (PF). Stain: Picro-Sirius under polarized light. Bar: 50 μm, x100. **(H’)** Calcified fibrocartilage (CF), rows of cells (arrows), bone (B). Stain: Picro-Sirius. Bar: 20 μm, x200. **(I’)** Calcified fibrocartilage (CF), bone (B), division between the collagen fibers of the tendon and bone (dashed line). Stain: Picro-Sirius under polarized light. Bar: 20 μm, x200. **(J)** Uncalcified fibrocartilage (UF), calcified fibrocartilage (CF), tidemark (dashed ellipse), bone (*). Stain: Hematoxylin–eosin. Bar: 50 μm, x100. **(J’)** Fibrocartilage cells in the uncalcified fibrocartilage (arrowhead), fibrocartilage cells in the calcified fibrocartilage (larger arrows), tidemark (small arrows). Stain: Hematoxylin–eosin. Bar: 20 μm, x200.

The histological samples stained with picrosirius red allowed the observation of the tendon tissue attached to the bone in both groups. The images show the adjacent periosteal fibrocartilage and the bone tissue with the trabecular spaces surrounded by trabecular tissue. (Fig 1A, 1C, 1F and 1H). In a higher magnification of the calcified fibrocartilage area it is possible to observe several rows of cells (Fig 1C’ and 1H’).

In Fig 1B, 1D, 1G and 1I these same regions are showed under polarized light with collagen type I stained in yellow and red and type III stained in green. One may notice the contrasting predominance of type I collagen in the adult group (Fig 1B and 1D) and of type III in the elderly group (Fig 1G and 1I). Also, the periosteal fibrocartilage was clearly distinguished from the bone tissue in the adult rats while in the older group this region is not well discernible (Fig 1B, 1D, 1G and 1I). The magnification of the bone-tendon junction under polarized light clearly reveals types I and III collagen and the interdigitation of the fibers that constitute the tendon arranged in parallel in a well organized fashion towards the bone that, in turn shows the collagen fibers distributed in a disordered way (Fig 1D’ and 1I’).

With the hematoxylin-eosin technique it is possible to demonstrate the uncalcified and calcified fibrocartilage separated by tidemark that constitute the bone-tendon junction in both groups (Fig 1E and 1J). In the adult group, one may notice the trabecular space surrounded by the trabecular bone and the tidemark between the fibrocartilage. This tidemark is thinner in the adult rats than in the older rats (Fig 1E and 1J). In a higher magnification, it is possible to observe rows of fibrocartilage cells (Fig 1E’ and 1J’) a double tidemark in the adult group (Fig 1E’) and a marked single line in the elderly group between fibrocartilages (Fig 1J’).

### Scanning Electron Microscopy

**Fig 2:**
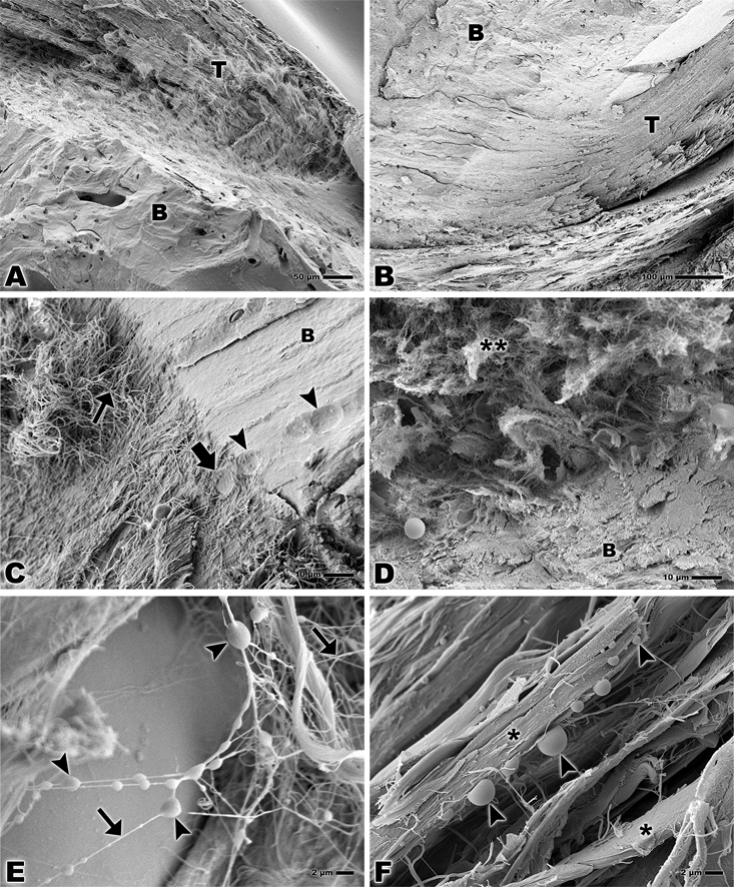
Scanning electron microscopy of bone-tendon junction of the calcaneal tendon of adults Wistar rats (A, C, E) and elderly Wistar rats (B, D, F). **(A, B)** Calcaneal tendon (T) and bone (B). Bar: 50 and 100 μm, magnification x200 and x160 respectively. **(C)** Collagen fiber of the tendon (small arrow), lacuna of fibrocartilage cell in the tendon tissue (larger arrow), lacuna of fibrocartilage cell in the bone (arrowheads), bone (B). Bar: 10 μm, x1,000. **(D)** Collagen fiber of the tendon (**) and bone (B). Bar: 10 μm, x1,000. **(E)** Lipid droplets (arrowheads), collagen fibers of the tendon tissue (arrows). Bar: 2 μm, x3,000. **(F)** Lipid droplets (arrowheads), bundles of collagen fibers of the tendon tissue (*). Bar: 2 μm, x3,000.

The SEM allowed the observation of the attachment of the calcaneal tendon to the bone tissue (Fig 2A and 2B). Magnification of the bone-tendon junction shows collagen fibers attaching to the bone and a higher degree of organization of the fibers in the adult group compared to the elderly rats (Fig 2C and 2D). In the adult group cell lacunae may be observed both in the collagen fibers of tendon and in the bone (Fig 2C).

Higher magnifications of tendon tissue show lipid droplets among the collagen fibers or bonding the fibers together (Fig 2E). These lipid droplets may also appear in collagen fibers bundles (Fig 2F).

**Fig 3:**
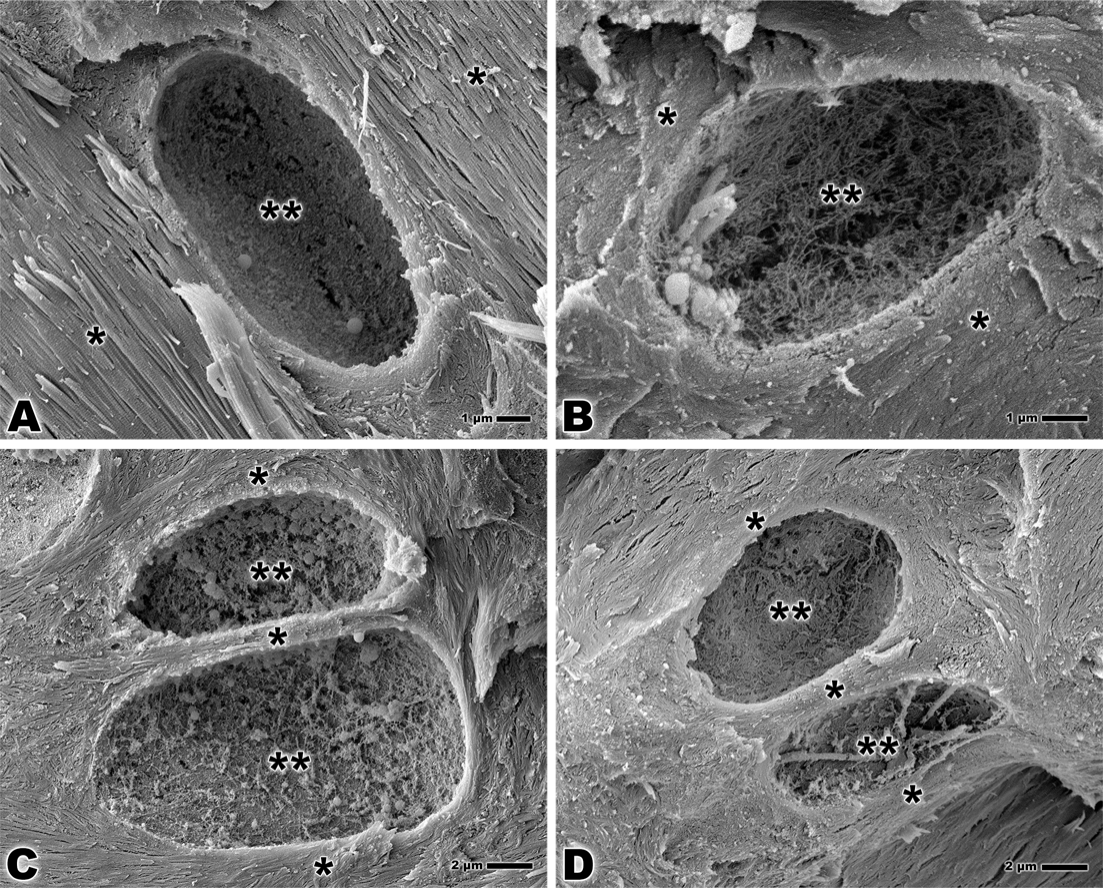
Scanning electron microscopy of bone-tendon junction of the calcaneal tendon of adults Wistar rats (A, C) and elderly Wistar rats (B, D). **(A, B)** Lacuna of fibrocartilage cell fibrocartilage (**), territorial matrix (*). Bar: 1 μm, x7,500 and x10,000. **(C, D)** Lacuna of fibrocartilage cell (**), territorial matrix (*). Bar: 2 μm, x5,000.

The analysis of the calcified fibrocartilage in both groups reveals a detailed morphology of the lacunae of fibrocartilage cells. In the lacunae, the collagen fibers intertwine to form a nest that has the similar size as the cell occupying it and the fibrocartilage may be seen around this complex (Fig 3A and 3B). The territorial matrix surrounding the lacunae may be seen in both groups (Fig 3C and 3D).

### Transmission Electron Microscopy

**Fig 4:**
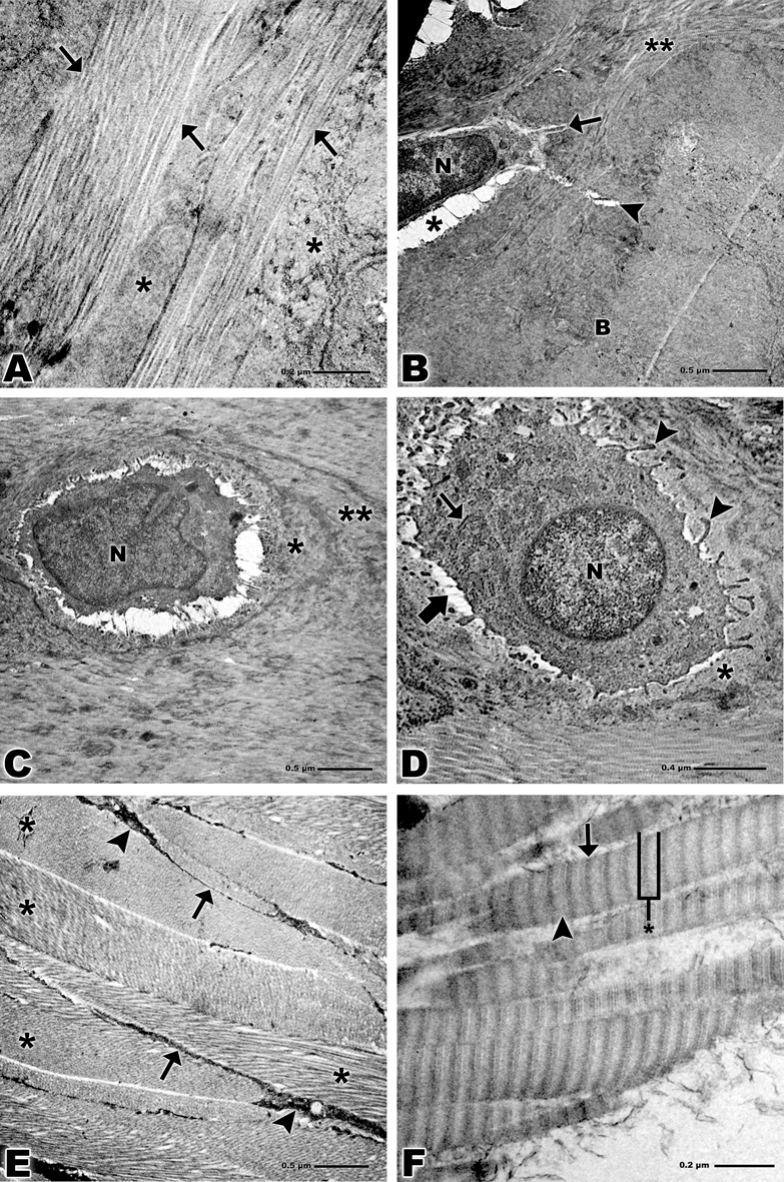
Transmission electron microscopy of bone-tendon junction of the calcaneal tendon of adults Wistar rats. **(A)** Collagen fibers of the tendon (arrows), calcified fibrocartilage (*). Bar: 0.2 μm, x15,000. **(B)** Collagen fibers (**), osteocyte nucleus (N), lacuna (*), cellular process (arrow), canaliculus (arrowhead), bone (B). Bar: 0.5 μm, x6,000. **(C)** Nucleus of fibrocartilage cell (N), territorial matrix (*), inter-territorial matrix (**). Bar: 0.5 μm, x6,000. **(D)** Nucleus of fibrocartilage cell (N), rough endoplasmic reticulum (small arrows), lacuna (larger arrow), cytoplasmic processes (arrowheads), territorial matrix (*). Bar: 0.4 μm, x10,000. **(E)** Bands of collagen fibers of the tendon tissue (*), tenocytes (arrowheads), cytoplasmic prolongation (arrows). Bar: 0.5 μm, x6,000. **(F)** Striations of the collagen fibrils of the tendon, overlap (arrow), gap (arrowhead) and overlap plus gap called the D-period (*). Bar: 0.2 μm, x94,000.

**Fig 5:**
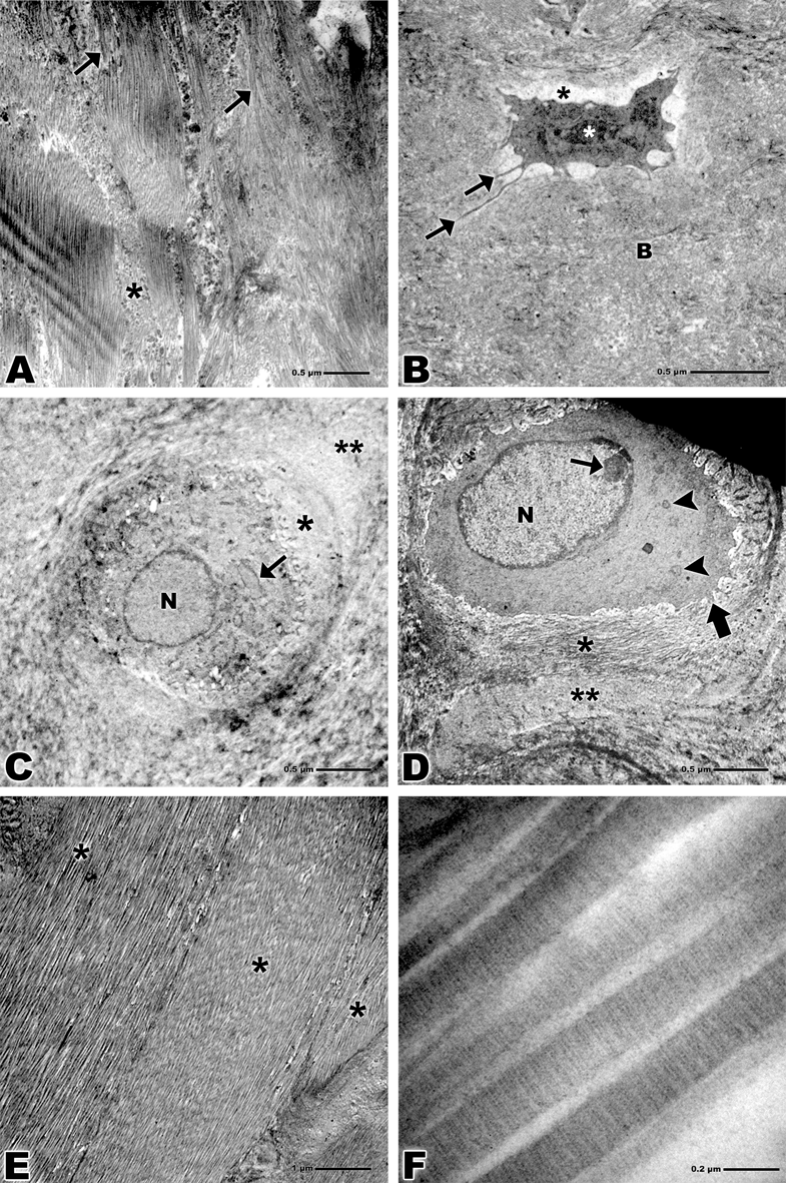
Transmission electron microscopy of bone-tendon junction of the calcaneal tendon of elderly Wistar rats. **(A)** Collagen fibers of the tendon (arrows), calcified fibrocartilage (*). Bar: 0.5 μm, x5,000. **(B)** Osteocyte nucleus (white asterisk), lacuna (black asterisk), cellular process (arrows), bone (B). Bar: 0.5 μm, x5,000. **(C)** Nucleus of fibrocartilage cell (N), rough endoplasmic reticulum (arrow), territorial matrix (*), inter-territorial matrix (**). Bar: 0.5 μm, x5,000. **(D)** Nucleus of fibrocartilage cell (N), nucleolus (small arrow), mitochondria (arrowheads), cytoplasmic processes (larger arrow), territorial matrix (*), inter-territorial matrix (**). Bar: 0.5 μm, x5,000. **(E)** Bands of collagen fibers of the tendon tissue (*). Bar: 1 μm, x3,000. **(F)** Striations of the collagen fibrils of the tendon, overlap (arrow), gap (arrowhead). Bar: 0.2 μm, x75,000.

TEM revealed collagen fibers attaching to the calcified fibrocartilage in an orderly fashion (Figs 4A and 5A). The osteocytes presented an elongated shape and extensive nucleus in the bone tissue and become lodged in the lacunae as well as the fibrocartilage cells. Their cellular processes traverse the interior of the canaliculi. Rounded collagen fibers and bone tissue were also observed (Figs 4B and 5B).

Fibrocartilage cells in both groups presented nuclei of varied formats, though in the adult group these cells present larger cytoplasmic processes and longer granular endoplasmic reticulum when compared to the elderly group. Territorial and inter-territorial matrix were evident in both groups, in the elderly group was more evident a nucleolus and some mitochondria (Figs 4C, 4D, 5C and 5D).

Analyzes of collagen that constitute tendon tissue revealed the collagen organized in bands with some tenocytes visible among these bands in the adult group (Figs 4E and 5E). At high magnification shows striations of the collagen fibrils of the tendon tissue, the dark bands are called overlap and the white bands gap, both constitute a space called D-period (Figs 4F and 5F).

### Type I and III of Collagen and Length of the D-Period of collagen fibrils Statistical Analysis

**Fig 6:**
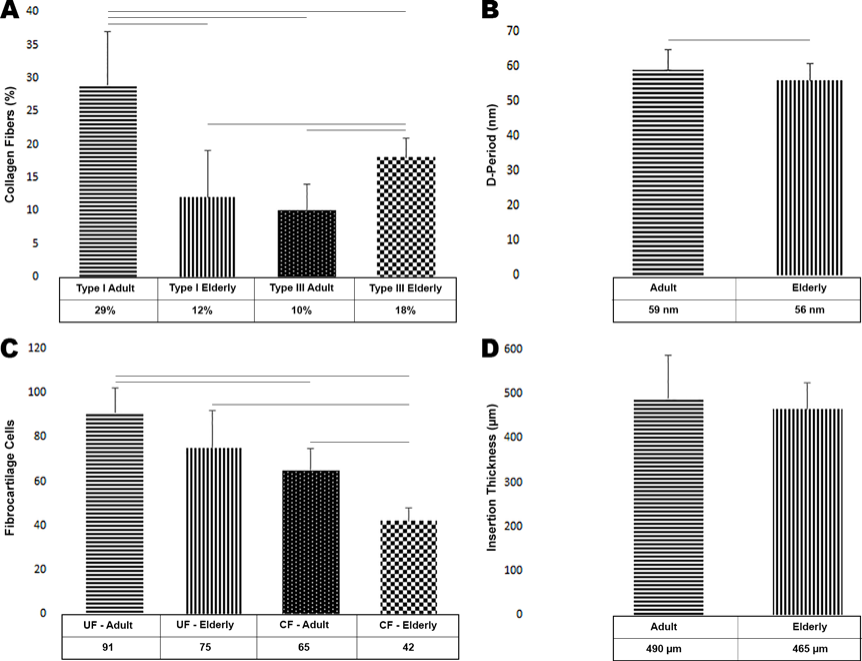
Quantitative analysis of collagen type I and III, D-period of the collagen fibers, fibrocartilage cells and insertion thickness of the calcaneal tendon into the bone. **(A)** Graph of collagen type I and III in the adult and elderly groups. **(B)** Graph D-period length of the collagen fibers. **(C)** Graph of fibrocartilage cells in uncalcified and calcified fibrocartilage region in the adult and elderly groups. **(D)** Graph of insertion thickness of the calcaneal tendon into bone tissue. In all graphs horizontal lines indicates statistical difference. Horizontal lines represent statistical difference.

The values obtained show an amount of 29 ± 8% of collagen type I in the adult group and 10 ± 4% of type III. This result shows statistical difference (*p* < 0.0001) enrichment of 65% of type I collagen compared to type III (Fig 6A). In contrast, the elderly group revealed an amount of 12 ± 7% type I collagen and of 18 ± 3% type III, an enrichment of 32% of type III compared to type I (*p* < 0.0166) (Fig 6A).

These results account for a difference of 57% in the concentration of collagen type I between the two groups, with a significant enrichment in the adult group (*p* < 0.0002) (Fig 6A). Regarding collagen type III, the elderly group shows a statistical difference (*p* < 0.0001) enrichment of 44% (Fig 6A).

When compared collagen type I in the adult group with the type III in the elderly group there is statistical difference (*p* < 0.0043), but when compared collagen type I in the elderly group with the type III in the adult group there is not statistical difference (*p* = 0.3639) (Fig 6A).

Analyzes of the D-period length of collagen fibrils showed an amount of 59 ± 6 nm in the adult group and 56 ± 5 nm in the elderly group, this difference is significant (*p* = 0.0173) (Fig 6B).

### Fibrocartilage Cells and Tendon Insertion Thickness Statistical Analysis

Our analysis in an area of the 0.15mm^2^ obtained 91 ± 11 fibrocartilage cells in the uncalcified fibrocartilage region and 65 ± 10 cells in the calcified fibrocartilage region in the adult group, an enrichment of 29% of cells in the uncalcified fibrocartilage region (*p* < 0.0001) (Fig 6C). In the elderly group, the uncalcified fibrocartilage region presented 75 ± 17 cells in contrast to 42 ± 6 cells in the calcified fibrocartilage, showing an enrichment of 44% in the uncalcified fibrocartilage region (*p* < 0.0022) (Fig 6C).

In the uncalcified fibrocartilage region the amount of fibrocartilage cells in the adult group was 91 ± 10 and 75 ± 17 in the elderly group, an enrichment of 18% of cells in the uncalcified fibrocartilage region in the adult group. This result, however, showed no statistical significance (*p* = 0.0605) (Fig 6C). In the calcified fibrocartilage region the amount of fibrocartilage cells of the adult group was 65 ± 10 and 42 ± 6 cells of the elderly group, 35% more cells in the adult group (*p* < 0.0001) (Fig 6C). However, when compared the cells of the uncalcified fibrocartilage in the adult group with the cells of the calcified fibrocartilage in the elderly group (*p* < 0.0001). On the other hand, when compared cells of the uncalcified fibrocartilage in the elderly group with cells of the calcified fibrocartilage region in the adult group there is not statistical difference (*p* = 0.2091) (Fig 6C).

The thickness of the calcaneal tendon insertion into the bone tissue was 490 ± 97 μm in the adult group and 465 ± 59 μm in the elderly group. This result shows a 5% thicker insertion of the adult rats and no statistical difference (*p* = 0.4804) (Fig 6D).

## Discussion

Our results showed the structural and ultrastructural morphological characteristics of the calcaneal bone-tendon junction and the alterations in collagen fibers and fibrocartilage cells of uncalcified and calcified regions that take place during aging in rats.

In both groups, adults and elderly, the analyzes of light microscopy revealed the tendon formed by cells arranged in rows separated by connective tissue. Bundles of this tissue compose the collagen fibers that constitute the basic unit of the tendon, that is the smallest unit visible by light microscopy [33].

We have demonstrated that tendon collagen fibers crossing fibrocartilaginous zones attaching into the bone, forming a deep interdigitation and an irregular border between lamellar bone and calcified fibrocartilage as suggested by Zhao and colleagues (2014) [34], these characteristics are a direct type of the enthesis.

Apparently, the depth of interdigitation decreases in the elderly group, thus we consider to be a possible cause of injury in older people.

However, analysis of the SEM demonstrated the interface between collagen fibers of the calcaneal tendon and bone in adult and elderly rats. This analysis method showed that the bone collagen fibers intertwined and merged with those of the tendon, similar result was obtained by Zhao and colleagues (2014) [34] which authors analyzed porcine anterior cruciate ligament tibial enthesis, thus we may suggest that both entheses present a similar organization of the collagen fibers.

Between uncalcified and calcified fibrocartilage may be seen the tidemark, the location where mineralization with apatite abruptly starts [35], in our study the adult rats presented a double tidemark that has been interpreted as a consequence of ‘start-stop’ phases of calcification [36], in the elderly rats only one wavy tidemark is observed, which suggests an advanced degree of mineralization.

Our study was realized in normal entheses, when it is compared with Milz and colleagues (2004) [37] which the authors evaluated histopathology of the entheses in middle aged cadavers (mean 47 years-old) and elderly cadavers (mean 84 years-old), both studies observed that fibrocartilage is a normal characteristics of all entheses from subjects of a wide age range that should not merely be equated with pathological change. However, Milz and colleagues (2004) [37] suggest mechanical overload may induce bony spur formation, in their study it was observed in the lateral epicondyle, whereas in our study was not observed in the region analyzed.

Furthermore, unlike from our study which was not observed inflammatory cells, the above authors mention the presence of this cells around small venules, and explain the majority of changes seen in elderly cadavers were degenerative rather than inflammatory and suggests that such degenerative changes are normal in elderly people.

Our observations of the fibrocartilage cells demonstrates a morphology rounded/elongated housed in the lacunae, revealing several cytoplasmic processes of different lengths. The cytoplasmic processes are detached in the territorial matrix, and the lacunae are formed when the collagen fibrils surround these processes forming interconnected three-dimensional structures [38].

Similarly to the fibrocartilage cells, the osteocytes present in the analyzed region are also lodged in the lacunae formed by a complex network of collagen fibers. The osteocytes extend cell processes within the canaliculi and communicate with neighboring cells. They have a dendritic morphology, and their cell body are fusiform in long bones or rounded in flat bones [39]. In general, lacunae and canaliculi of the lacuna-canalicular network are surrounded by bone matrix [40]. Lacunae contain the cell bodies with slender cytoplasmic processes rich in actin (each cell has 50–60 cellular processes) that propagate through the canaliculi [41], lacunae did not differ with age, as well as collagen network did not present any significant alterations observed by used methods.

The ultrastructural analysis revealed that the adult cells contain more developed organelles, however, both groups showed highly variable nuclei formats. The nuclei are typically rounded, but multilobulated or jagged shaped are also frequent [42].

Functional changes during aging reduce the synthetic capacity of cells, that peaks ​​in newborns and young animals and reaches its minimum in the elderly [43–45]. The decreased synthesis caused by aging is related to the degradation of the Golgi apparatus, and the granular endoplasmic reticulum and scarcity of mitochondria [42].

Our analyzes also showed the presence of lipid droplets among the collagen fibers of the both groups. The number of lipid droplets increases with increasing age and especially under pathological conditions. However, the exclusive presence of small lipid droplets does not cause structural or chemical anomalies in collagen fibers and, therefore, is unlikely to affect the biomechanical properties of tendons [46].

High magnification images noted by TEM showed clearly the striations that constitute the collagen fibrils. Those striations are formed by dark bands called the overlap contains five molecules and by white bands called the gap and contains only four molecules, both bands together are called the D-period.

Thus the D-period present a periodicity of about 70 nm based on a staggered arrangement of individual collagen monomers [47], however, we observed a D-period about 59 nm in the adult groups and 56 nm in the elderly group. The cited authors do not inform which animal model like reference.

Moreover, Lavagnino and colleagues (2013) [48] demonstrate in their study a shortening of the tendon as a age-related phenomenon affecting the contraction capacity. Our result show a lesser D-period in the elderly group which suggest a shortening of the collagen fibrils.

Macroscopically our results showed no changes in bone-tendon junction, however, at the microscopic level, the number of cells present in the regions of fibrocartilage was strikingly different. In both regions (uncalcified and calcified), the number of cells was higher in adult rats, but no statistical difference was observed regarding the insertion thickness between the groups.

To analysis types of collagen we used a histological quantification method using glass slides with picrosirius red staining analyzed under polarized light, this method differs type of collagen by color, yellow-red strong birefringence would be assigned to collagen type I and collagen type III would display a weak birefringence associated with a greenish color [49].

Constantine and Mowry (1968) [50] explain that collagen has a natural birefringence which is attributed to the arrangement of its fibers. Picrosirius red staining is the better method to collagen, because consistently stain thin collagen fibers, did not fade, and is appropriate for use with polarized light microscopy [51].

Lattouf and colleagues (2014) [21] conclude their study stating picrosirius red analyzed under polarized light is simple, sensitive and specific for collagen staining, it is particularly useful to reveal the molecular order, organization and/or heterogeneity of collagen fiber orientation in different connective tissues and this method may be a precious tool in measuring the amount of collagen content in normal or pathological tissues.

As well as in our study other authors has used this method in various tissues to quantify collagen, for example Singh and colleagues (2012) [52] in their study about role of connective tissue stroma on biological behavior of odontogenic cysts, Geary and colleagues (2015) [53] in study of tendon repair, Manjunatha and colleagues (2015) [54] in oral squamous cell carcinoma, Alves and colleagues (2015) [55] in a study of the splenic collagen polymorphism, and many others.

Our statistical analysis about proportion of collagen type III relative to type I suggest that type III increases with age.

The collagen type I fibrils are resistant structures that provide strength and mechanical durability to the tendon tissue while type III is constituted by thinner fibrils and has an important role in the healing process, but presents a reduced resistance to mechanical stress [56–59].

Although Yu and colleagues (2013) [60] explain aging affects the enzymatic activities of MMP-2 and -9 and their physiologic inhibitors TIMP-1 and -2 directly, in their study the authors reported that these metalloproteinases mRNA expressions increases significantly with age. Furthermore, as compared with young rats, mRNAs that encode TIMP-1 and -2 are decreased in fibroblast present in the tendon (tenocytes) from the elderly rats and conclude that activities of both MMP-2 and -9 are higher in the tendons of aging rats than in the tendons of young rats. Regarding TIMP-1 and -2, their results reveal that both mRNA expression decrease in old tenocytes, under less inhibitory effect from TIMP-1 and -2, may further have a more negative impact on the integrity of tendon matrix. On the other hand, analyzes of RT-qPCR realized by Kostrominova and Brooks (2013) [61] showed 28.5-fold decreases in COL1a1 (collagen type I gene) mRNA expression and 7.3-fold decreases in COL3a1 (collagen type III gene) mRNA. Despite significant decreases in both genes mRNA expression in calcaneal tendon of elderly rats, relative levels of protein expression based on intensity of immunostaining showed almost 2.5 times more COL3a1 than COL1a1 in elderly rats compared with young rats.

This result of protein expression coincides with ours and suggest that both collagens decreases, however, type I decreases more than the type III, occurring a predominance of collagen type III in the elderly group.

Consequently, the predominance of collagen type III is usually related to a decrease in the tendon’s resistance to tensile forces and may predispose to spontaneous tendon rupture [56,62].

These changes in collagen patterns also occur in the bone tissue. During aging, this process causes a deterioration in the stiffness of the bone matrix and structural changes in micro and nanometer scales [63]. The changes in the integrity of the collagen network may result in weaker bridges and, as a consequence, diminished resistance to fractures [63,64].

Although this study does not encompasses all aspects of the bone-tendon junction research the exploration of different approach methods may bring valuable insights about the morphological mechanisms of the tendon and their aging-related changes.

Scanning and transmission electron microscopy are methods of microanalysis rarely found in the literature to analyze the bone-tendon junction, and they both may properly provide a correlation in order to give a whole structural approach for the knowledge about the region studied.

## Conclusion

Aging-related changes in the bone-tendon junction may be too subtle to be identified macroscopically. Thickness of the calcaneal tendon insertion into the bone was similar in both groups. However, structural and ultrastructural analyzes may reveal important signs of degradation.

We showed a decrease of fibrocartilage cells in the elderly rats, both in calcified and uncalcified fibrocartilage. Also, we observed a predominance of collagen type III in this group compared to the adult rats, these two factors showed significant differences and may be related to a greater risk of injury. D-period length of the collagen fibers was measured and revealed to be lesser in the elderly group.

The analysis of scanning electron microscopy revealed the morphological aspects of the fibrocartilage cells lacunae, and the presence of the lipid droplets among the collagen fibers of the tendon in both groups; however, these droplets are not indicative of pathology.

The ultrastructural analysis realized by transmission electron microscopy showed fibrocartilage cells with shorter and fewer cytoplasmic processes and a reduced synthetic capacity due to smaller Golgi apparatus, fewer granular endoplasmic reticulum cisterns and fewer mitochondria in the elderly group when compared with adult rats. No difference was observed regarding size and shape of the nucleus in the two groups.

## Supporting Information

S1 Fig Macroscopic view from region analyzed to light microscopy and SEM (black dashed rectangle). To TEM (red dashed rectangle), tendon (T) calcaneal bone (C).
(TIF)

S2 Fig Light microscopy showing location which collagen types was quantify (dashed rectangle). Stain: Picro-Sirius under polarized light. Bar: 50 μm, x100.
(JPG)

S3 Fig Light microscopy showing location of fibrocartilage cells count in uncalcified (UF) and calcified (CF) fibrocartilage regions. Stain: Hematoxylin–eosin. Bar: 50 μm, x100.
(TIF)

S4 Fig Light microscopy showing the location where the insertion thickness of the tendon was measured. Stain: Hematoxylin–eosin. Bar: 50 μm, x100.
(TIF)
