# Supplementary figures and images for: Structural and Ultrastructural Characteristics of Bone-Tendon Junction of the Calcaneal Tendon of Adult and Elderly Wistar Rats
Source: PLoS One. 2016 Apr 14;11(4):e0153568. doi: 10.1371/journal.pone.0153568 (PMC4831835; doi:10.1371/journal.pone.0153568)

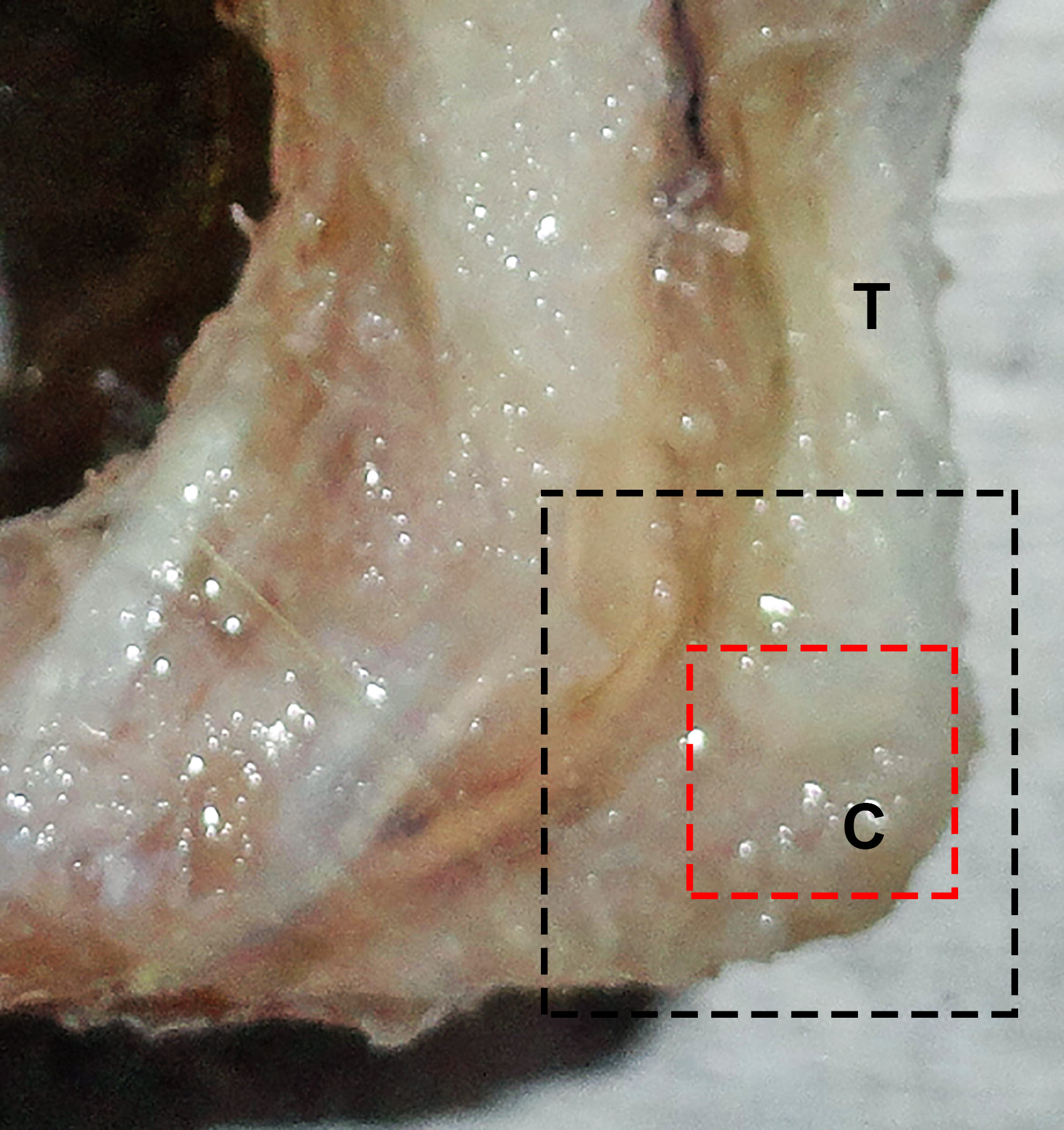

Supplement: S1 Fig — To TEM (red dashed rectangle), tendon (T) calcaneal bone (C). (TIF) [file pone.0153568.s001.tif]

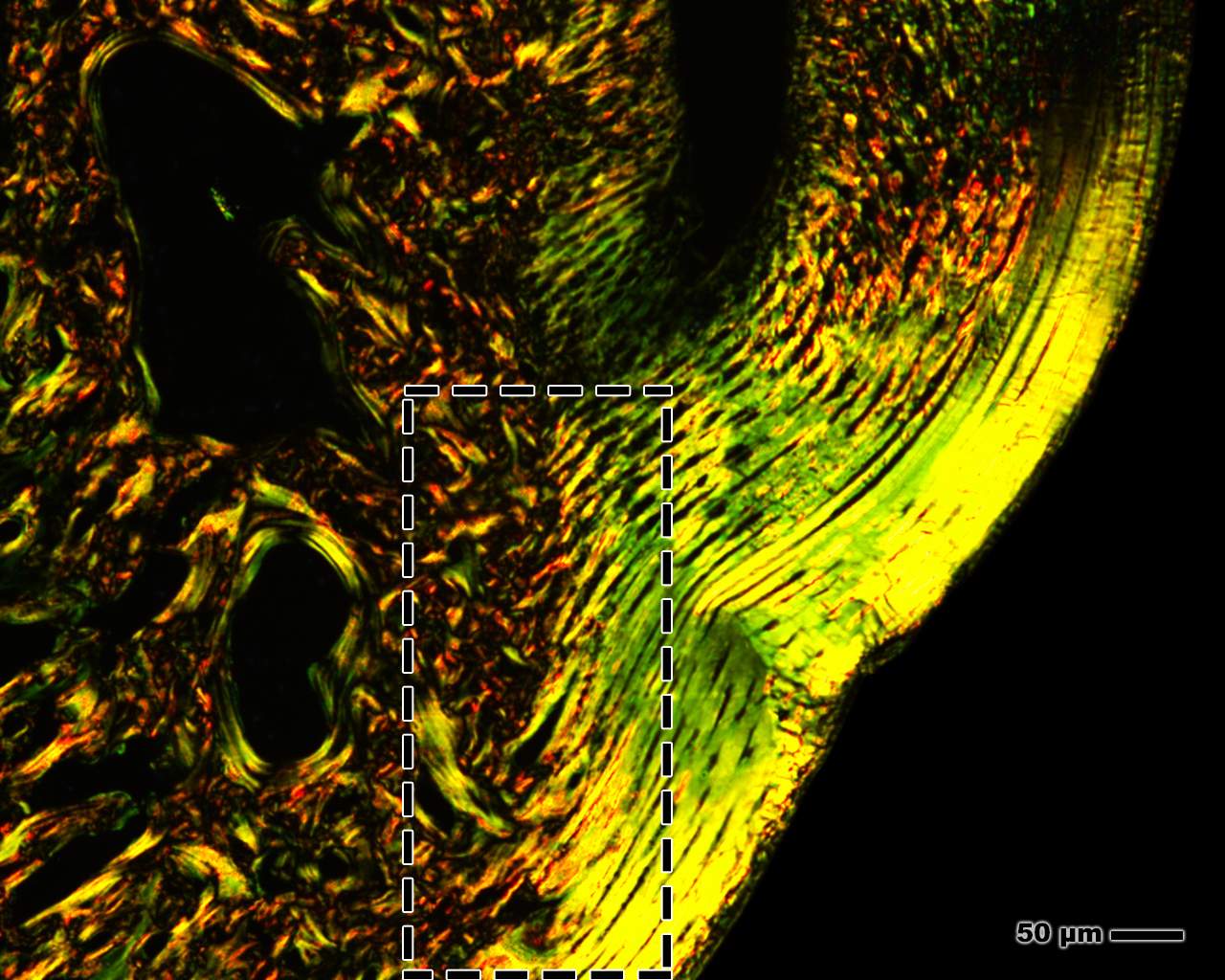

Supplement: S2 Fig — Stain: Picro-Sirius under polarized light. Bar: 50 μm, x100. (JPG) [file pone.0153568.s002.jpg]

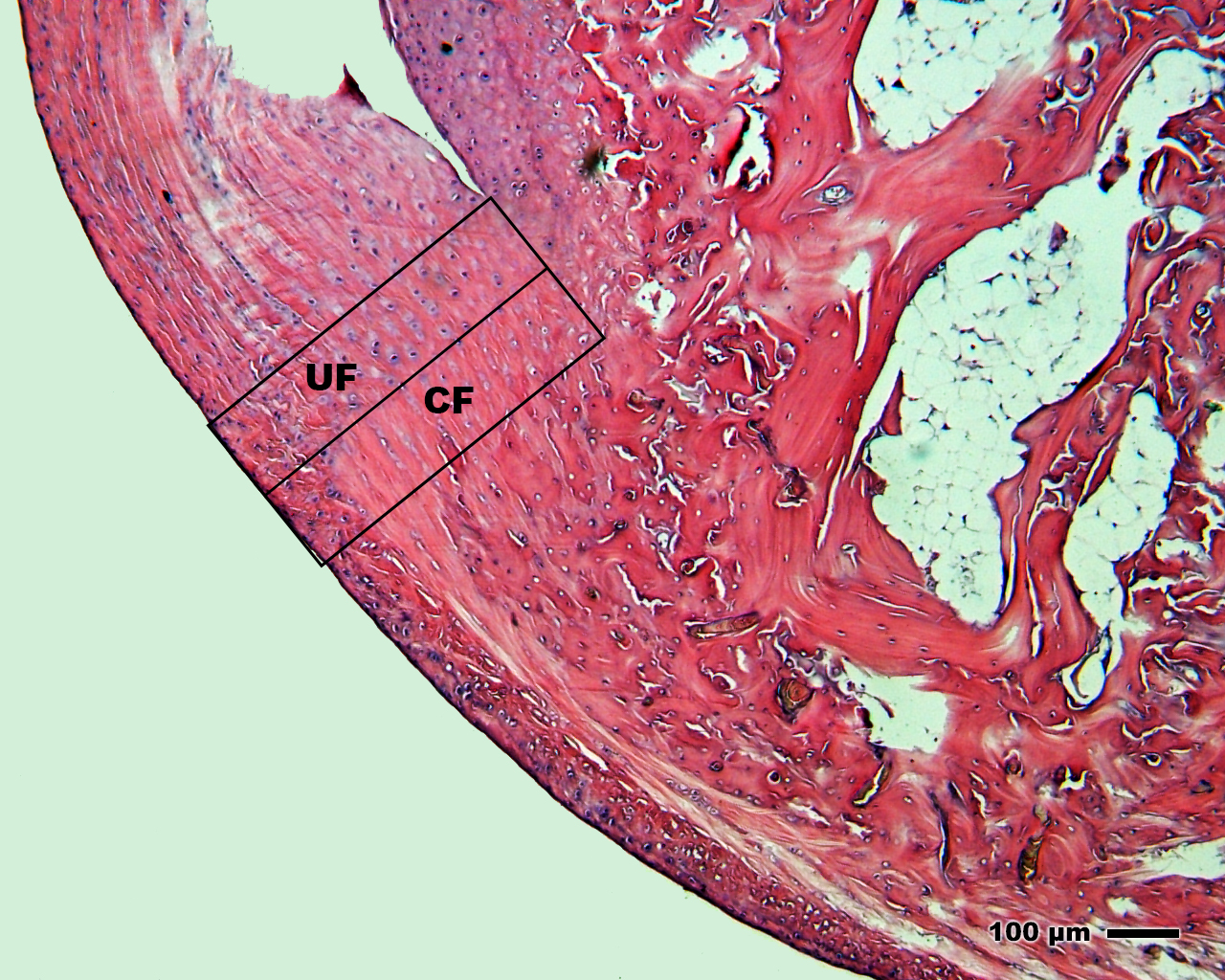

Supplement: S3 Fig — Stain: Hematoxylin–eosin. Bar: 50 μm, x100. (TIF) [file pone.0153568.s003.tif]

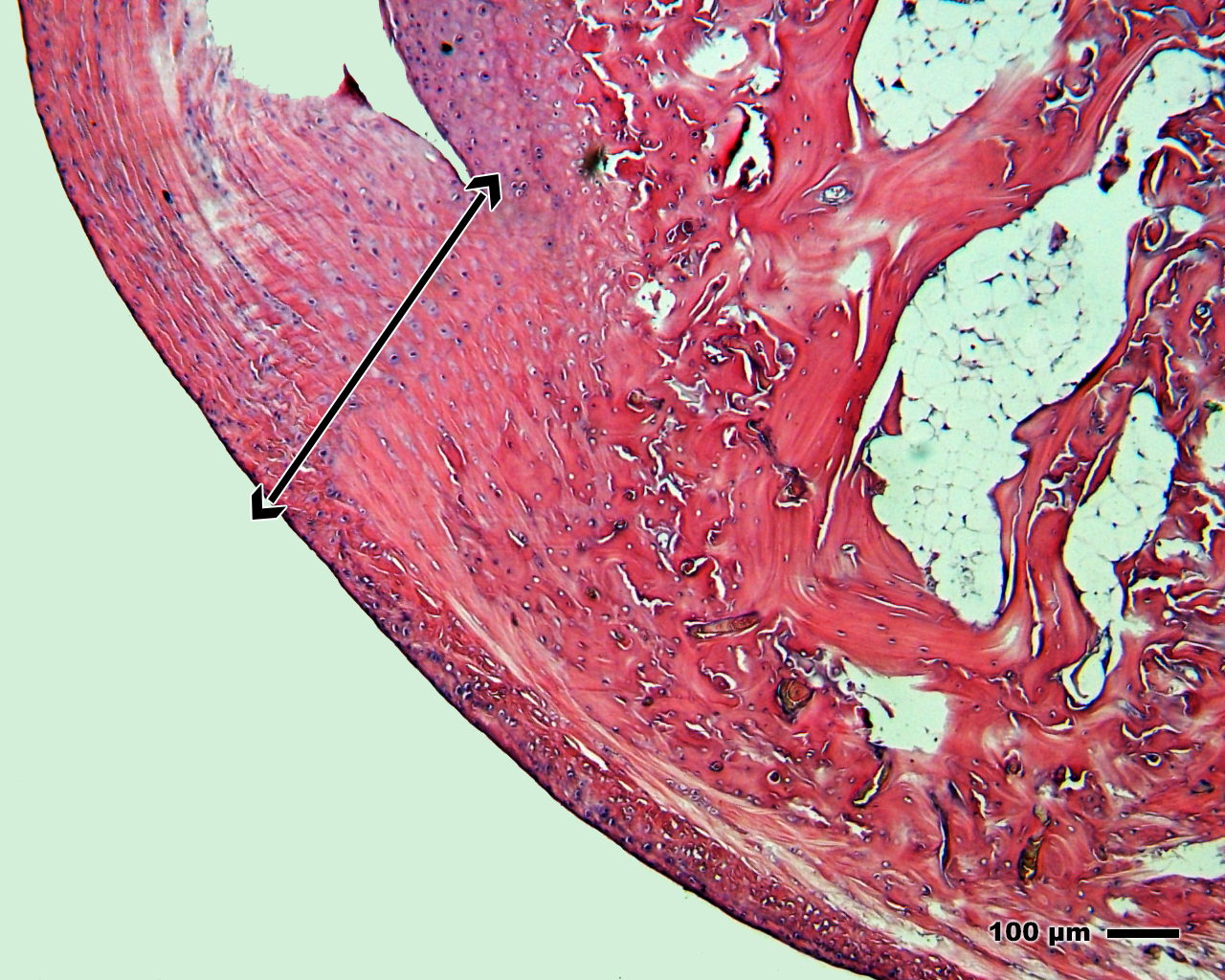

Supplement: S4 Fig — Stain: Hematoxylin–eosin. Bar: 50 μm, x100. (TIF) [file pone.0153568.s004.tif]
